# Recent advances in the diagnosis and management of amyloid cardiomyopathy

**DOI:** 10.12703/r/10-31

**Published:** 2021-03-24

**Authors:** Petra Nijst, WH Wilson Tang

**Affiliations:** 1 Department of Cardiology, Ziekenhuis Oost-Limburg, Genk, Belgium; 2 Biomedical Research Institute, Faculty of Medicine and Life Sciences, Hasselt University, Diepenbeek, Belgium; 3 Department of Cardiovascular Medicine, Heart and Vascular Institute, Cleveland Clinic, Cleveland, OH, USA

**Keywords:** cardiac amyloidosis, light chain amyloidosis, transthyretin amyloidosis

## Abstract

Amyloidosis is a disorder characterized by misfolded precursor proteins that form depositions of fibrillar aggregates with an abnormal cross-beta-sheet conformation, known as amyloid, in the extracellular space of several tissues. Although there are more than 30 known amyloidogenic proteins, both hereditary and non-hereditary, cardiac amyloidosis (CA) typically arises from either misfolded transthyretin (ATTR amyloidosis) or immunoglobulin light-chain aggregation (AL amyloidosis). Its prevalence is more common than previously thought, especially among patients with heart failure and preserved ejection fraction (HFpEF) and aortic stenosis. If there is a clinical suspicion of CA, focused echocardiography, laboratory screening for the presence of a monoclonal protein (serum and urinary electrophoresis with immunofixation and serum free light-chain ratio), and cardiac scintigraphy with ^99m^technetium-labeled bone-tracers are sensitive and specific initial diagnostic tests. In some cases, more advanced/invasive techniques are necessary and, in the last several years, treatment options for both AL CA and ATTR CA have rapidly expanded. It is important to note that the aims of therapy are different. Systemic AL amyloidosis requires treatment targeted against the abnormal plasma cell clone, whereas therapy for ATTR CA must be targeted to the production and stabilization of the TTR molecule. It is likely that a multistep treatment approach will be optimal for both AL CA and ATTR CA. Additionally, treatment of CA includes the management of restrictive cardiomyopathy with preserved or reduced ejection fraction in addition to treating the amyloid deposition. Future studies are necessary to define optimal management strategies for AL CA and ATTR CA and confirm cardiac response to therapy.

## Introduction

**Figure 1.**
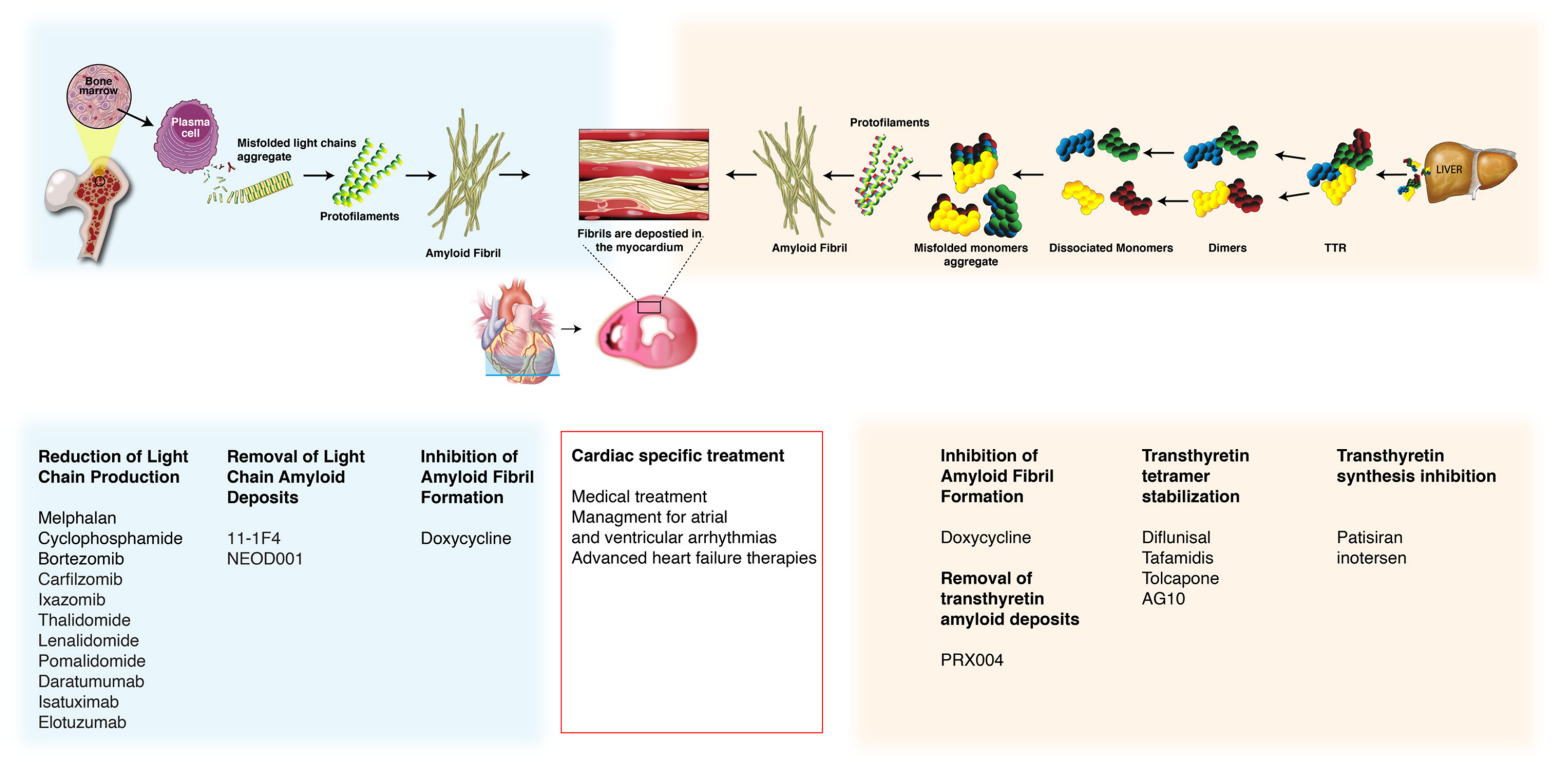
Cardiac amyloidosis: pathogenesis and treatment targets. The two main types of amyloidosis that affect the heart are immunoglobulin light-chain (AL) amyloidosis (blue), which results from aberrant plasma cell production of monoclonal light-chains, and transthyretin (TTR) amyloidosis (ATTR; orange), resulting from TTR produced by the liver. Misfolded proteins aggregate into amyloid fibrils that deposit extracellularly in the interstitial space of the myocardium. Different treatments and their targets in the pathogenesis of cardiac amyloidosis are presented.

Amyloidosis is characterized by organ dysfunction as a result of deposition of misfolded precursor proteins that form cross-beta-sheet amyloid fibrils in the extracellular spaces. Originally identified via pathology samples, amyloid fibrils’ affinity for Congo Red staining produces a pathognomonic “apple-green” birefringence when visualized under polarized light microscopy. Amyloid fibrils consist of proteins such as serum amyloid P (SAP), glycosaminoglycans, and calcium. There are more than 30 known amyloidogenic proteins (hereditary and non-hereditary), but cardiac amyloidosis (CA) arises from just two major causes: 1) immunoglobulin light-chain aggregation or 2) misfolded transthyretin (TTR)^1^ (Figure 1). Other amyloid types, including amyloid A, apolipoprotein AI, heavy chain, and atrial natriuretic peptide, can also involve the heart but they are extremely rare. Amyloid light-chain (AL) amyloidosis results from misfolding of overproduced immunoglobulin light-chains (kappa or lambda) secreted by abnormal plasma cells such as those in multiple myeloma. It is a rare disease, with about 3,000 new cases per year in the United States, but the heart is involved in 50–75% of these cases^2^. The other major cause of CA is the misfolding of TTR (formerly named pre-albumin), which is produced by the liver and serves as a transport protein for thyroxine and other proteins. The TTR protein contains four identical subunits rich in beta-sheets that assemble into a tetramer as two associated dimers. Formation of TTR amyloid fibril requires the dissociation of the tetramer into alternatively folded monomers, which then self-assemble to form insoluble amyloid fibrils^3^. TTR amyloidosis can be due to wild-type (ATTRwt, no mutation, previously called “senile cardiac amyloidosis”) or a hereditary genetic variant of TTR (ATTRm), of which there are currently more than 100 described. It is not well understood why ATTRwt proteins become kinetically unstable and aggregate, but it appears to involve the aging process^4^. In fact, up to 25% of patients 85 years and older show ATTRwt amyloid deposits on autopsy studies^5^. In addition, ATTRwt is much more common than previously thought and is likely underdiagnosed, particularly among patients with HFpEF and calcific aortic stenosis^6,7^. It is therefore conceivable (but speculative) that pressure loading may promote myocardial TTR deposition. Another possible etiologic mechanism is that the inflammatory processes and oxidative stress associated with calcified aortic stenosis may promote amyloid deposition^8,9^.

## Natural history and prognosis of cardiac amyloidosis

In general, ATTR CA is characterized by years of relative clinical stability despite advanced disease shown by imaging, hemodynamics, and reduced functional capacity. This is commonly followed by a slow but steady decline that transitions to severe and refractory HF. Accordingly, patients with ATTR CA often “look much better” clinically than cardiac imaging and invasive hemodynamics would suggest^4^. This is in contrast to AL amyloidosis, where the imaging findings may be subtle despite rapidly progressive HF^10^. The discrepancy has been attributed to the direct cardiotoxicity of circulating free light-chains (FLCs) and pre-fibrillar aggregates in CA caused by AL amyloidosis^2,4^.

The presence and extent of cardiac involvement is a major determinant of outcome in both AL amyloidosis and ATTR amyloidosis. Despite AL amyloidosis being a disease that fundamentally arises as a result of a hematologic malignancy, the prognosis is primarily driven by the extent of cardiac involvement. The median untreated survival is less than 6 months in patients who present with AL CA and HF^11^. A validated staging system can be used to predict 5-year survival of CA caused by AL amyloidosis based on the presence of elevated levels of biomarkers such as cardiac troponin, natriuretic peptides, and FLC ratio (Table 1). Prognosis has markedly improved in AL amyloidosis over the last two decades. With current treatment regimens, patients in all but the highest risk group have a median survival of >4 years and some groups have a median survival of >10 years^12^. However, median survival in stage 4 patients remains poor (around 6 months)^13^. In contrast, the median survival of untreated patients diagnosed with ATTRm is 2–3 years but depends on the underlying genetic mutation and is around 4 years for ATTRwt^4,14^ (Table 1).

**Table 1.** Amyloidosis staging systems and risk stratification.

Mayo AL Amyloidosis Staging^23^	Mayo ATTRwt CA Staging^24^	NAC ATTR CA Staging^14,25^
**Prognostic variables:** • cTnT ≥0.025 ng/ml = 1 point • NT-proBNP ≥1,800 pg/ml = 1 point • FLC-diff ≥18 mg/dL = 1 point	**Prognostic variables:** • cTnT ≥0.05 ng/ml = 1 point • NT-proBNP ≥3,000 pg/ml = 1 point	**Prognostic variables:** • NT-proBNP ≥3,000 pg/ml = 1 point • eGFR <45 ml/min/1.73 m^2^ = 1 point
**5-year survival estimate** Stage I (0 points): 59% Stage II (1 point): 42% Stage III (2 points): 20% Stage IV (3 points): 14%	**4-year survival estimate** Stage I (0 points): 57% Stage II (1 point): 42% Stage III (2 points): 18%	**5-year survival estimate** Stage I (0 points): 56–62% Stage II (1 point): 22–38% Stage III (2 points): 12–23%

Abbreviations: ATTR, transthyretin amyloidosis; ATTRwt, wild-type transthyretin amyloidosis; CA, cardiac amyloidosis; cTnT, cardiac troponin T; eGFR, estimated glomerular filtration rate; FLC-diff, free light-chain difference; NAC, National Amyloidosis Center; NT-proBNP, N-terminal of the prohormone of B-type natriuretic peptide

## Advances in the diagnosis of cardiac amyloidosis

**Figure 2.**
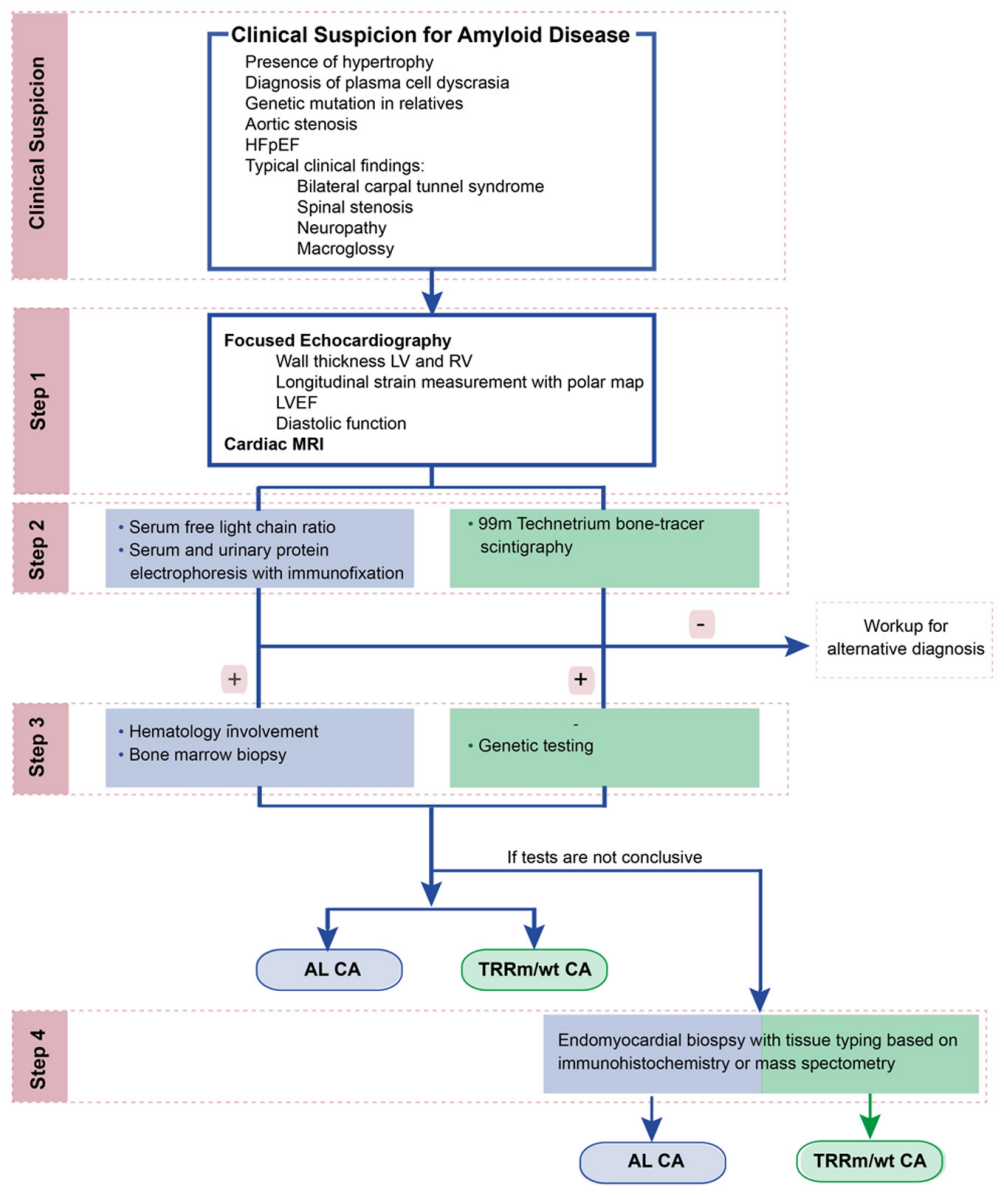
Diagnostic algorithm for the diagnosis of CA. AL, amyloid light-chain; ATTRm, hereditary (or mutant) transthyretin amyloidosis; ATTRwt, wild-type transthyretin amyloidosis; CA, cardiac amyloidosis; HFpEF, heart failure with preserved ejection fraction; LV, left ventricle; LVEF, left ventricular ejection fraction; MRI, magnetic resonance imaging; RV, right ventricle.

Early diagnosis of CA is crucial, since mortality is high without prompt recognition and treatment. Figure 2 gives a general overview of the diagnostic algorithm for CA used in our center, which is comparable to previously published algorithms^15^. Often a workup for CA starts after a clinical suspicion of CA, which may include the presence of unexplained ventricular hypertrophy or stiffness on echocardiography, the presence of a non-specific cardiac magnetic resonance imaging (MRI) pattern of late gadolinium enhancement (LGE) suspicious of CA, the diagnosis of a plasma cell dyscrasia, the diagnosis of a genetic variant in a family member, or an antecedent medical history suggestive of an infiltrative disease. For example, bilateral carpal tunnel syndrome is predominantly present in patients with ATTR amyloidosis and can precede heart symptoms by 5–15 years^14,16^. Spinal stenosis is common in patients with ATTRwt variant and is due to amyloid infiltration in the ligamentum flavum^17^. Peripheral and autonomic neuropathy can occur in both AL and ATTRm amyloidosis but are uncommon in ATTRwt amyloidosis^18^ (Table 2).

**Table 2.** Typical characteristics for AL and ATTR CA.

	AL CA	ATTR CA
**Age of onset**	Median age 63 years	≥60 years
**Gender**		Male
**Race**		White (T60A mutation, Irish descent) African American (V122I mutation)
**Common clinical** **symptoms**	Atrial fibrillation/flutter	Atrial fibrillation/flutter
	Carpal tunnel	(Bilateral) carpal tunnel
		Spinal stenosis
	Peripheral neuropathy	Peripheral neuropathy
	Heart block and/or bundle branch block	Heart block and/or bundle branch block
	Low flow, low grade aortic stenosis	Low flow, low grade aortic stenosis
	Hypertrophic CMP Low voltage ECG (overall degree of voltage relative to the degree of LV thickening)	Hypertrophic CMP in older age Low voltage ECG (overall degree of voltage relative to the degree of LV thickening)
	Renal (nephrotic syndrome), GI symptoms	Distal biceps tendon rupture
	Macroglossia	
	Periorbital purpura	
**Median survival**	1–3 years	2–6 years
**Therapy**	Combination chemotherapy (alkylating agents, proteasome inhibitors, steroids) Monoclonal antibodies Immunomodulatory therapy Autologous stem cell transplant Heart transplant	siRNA TTR stabilizer Amyloid fibril disruptor Heart transplant Combined heart and liver transplant

Abbreviations: AL, amyloid light-chain; ATTR, transthyretin amyloidosis; CA, cardiac amyloidosis; CMP, cardiomyopathy; ECG, electrocardiogram; GI, gastrointestinal; LV, left ventricle; siRNA, small interfering RNA; TTR, transthyretin

### Echocardiography

**Figure 3.**
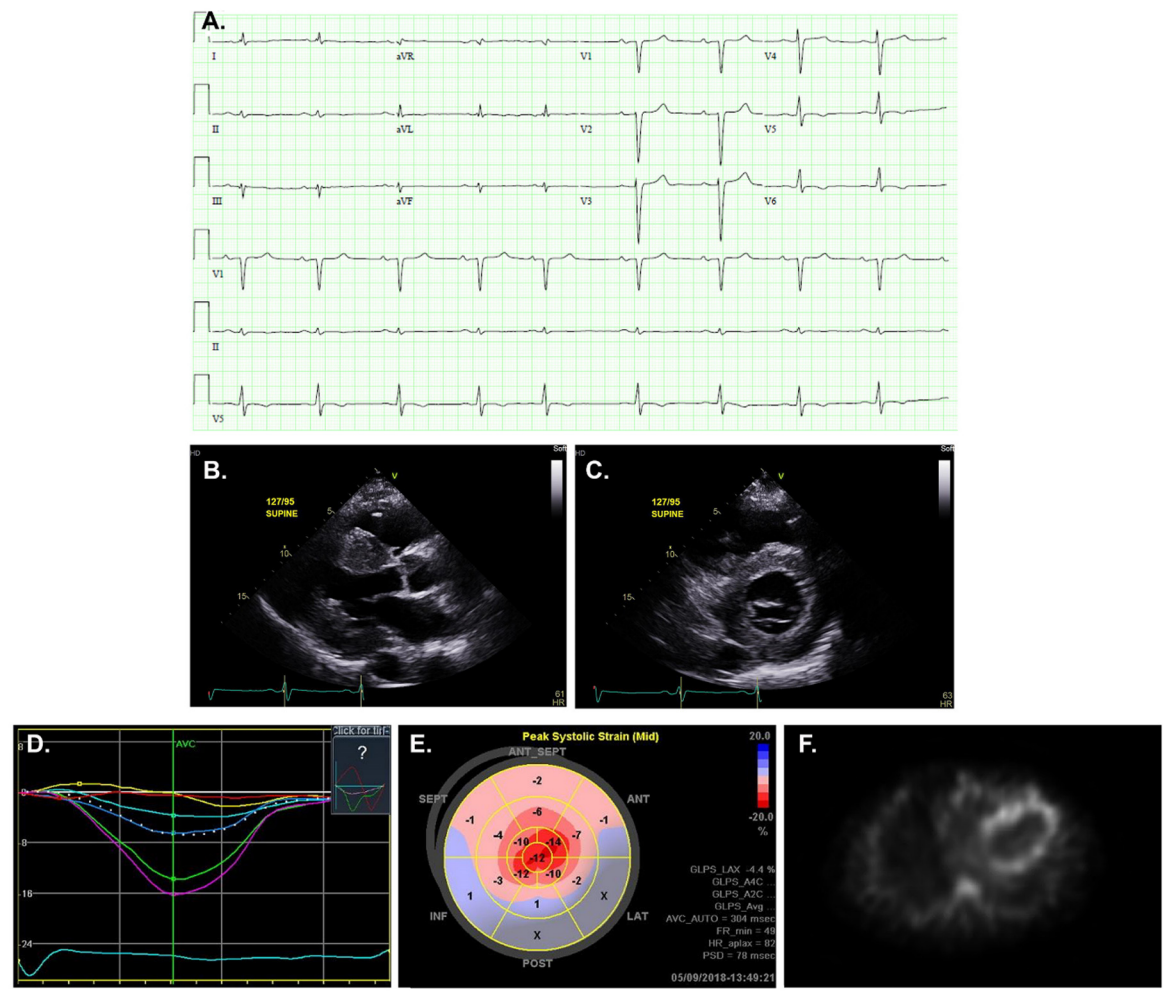
Diagnostic tests for cardiac amyloidosis. (**A**) Electrocardiogram showing characteristic low voltages relative to left ventricle wall thickness, with a pseudo-infarct pattern with Q waves in the early precordial leads mimicking a prior anteroseptal myocardial infarction. (**B** and **C**) Transthoracic echocardiogram, parasternal long-axis and short-axis view, showing increased ventricular wall thickness and thickening of the mitral valve. (**D** and **E**) Longitudinal strain imaging using two-dimensional speckle tracking echocardiography reveals the characteristic bull’s-eye pattern of apical sparing. (**F**) ^99m^Technetium pyrophosphate scan shows grade 3 myocardial radiotracer uptake characteristic of transthyretin cardiac amyloidosis.

The appropriate next step in diagnosing CA is a focused echocardiogram if not already performed or, in case of insufficient image quality, a cardiac MRI (Figure 3). Typical findings are left ventricular (LV) wall thickness >12 mm, the presence of right ventricular (RV) hypertrophy, and abnormalities of diastolic function or the presence of restrictive physiology. Longitudinal strain imaging is used to measure the actual deformation of myocardium in specific LV segments, and quantification is displayed as a polar map. Several distinguishing characteristics of CA can be seen on echocardiogram. For example, apical sparing, in which the apical LV segments have normal or near-normal strain compared with the mid and basal segments, is commonly seen in CA. The easily recognizable bull’s-eye (“cherry-on-top”) pattern on the polar map can help differentiate CA from other forms of LV hypertrophy with good sensitivity and specificity^19^. Historically, the characteristic myocardial “sparkling” pattern has low sensitivity and specificity and should not be relied on^18^. Although LV ejection fraction is usually preserved in CA, cardiac output is low because of decreased ventricular volume^20^. Another important echocardiographic clue that can differentiate CA from other diseases is the presence of biventricular and concomitant valvular thickening, which is not often seen in hypertensive heart disease of hypertrophic cardiomyopathy^21^. A final classic hallmark of CA to look for is the combination of low voltage on electrocardiogram (ECG) and increased LV wall thickness on echocardiogram^22^.

In the future, the diagnostic accuracy of echocardiography will likely increase by combining several parameters. A recent study showed that amyloidosis can be confirmed or excluded on the basis of echocardiogram scores in about 50% of cases^26^. These scores include wall thickness, E/e’ ratio, longitudinal strain, and tricuspid annular plane excursion. In another study, Zhang and coauthors investigated the potential of automated cardiac image interpretation for the detection of CA and other cardiac diseases and demonstrated that deep machine learning models may help to automatically detect these diseases in large healthcare imaging systems^27^. At this point, however, despite increasing interest, future studies are necessary to understand how machine learning can be used to improve the diagnostics of CA.

### Cardiac magnetic resonance imaging

Cardiac MRI is useful for the diagnosis of CA. In addition to the typical structural abnormalities in CA seen with echocardiography, global subendocardial enhancement and transmural LGE or focal patchy LGE are typical MRI features of CA^28^. Traditional LGE imaging techniques require an operator-determined null point, which is defined as the inversion recovery time at which the normal myocardium appears black. This can be challenging in CA, and difficulty nulling is strongly suggestive of CA with sensitivity of 100%^29^. Additionally, cardiac MRI can reveal the characteristic LGE pattern that is diffuse and subendocardial without following a coronary artery distribution. The LGE pattern can also be seen in the right ventricle, and even the atrial walls, and can be transmural and patchy in ATTRwt CA. This LGE pattern is highly sensitive (93%) and specific (70%) for CA with an overall negative predictive accuracy of 84%^29^. Of note, this high negative predictive value of cardiac MRI makes this test very interesting to screen asymptomatic family members of patients with genetic forms of amyloidosis. In addition, CA patients with transmural LGE have a fivefold increase in mortality when compared to with those without LGE^29^. In addition to LGE, native (pre-gadolinium contrast) T1-myocardial mapping techniques, which provide a combined signal from myocyte and extracellular space, are also useful in CA diagnosis. Significantly increased native T1 times are seen in CA and have been associated with outcomes in patients with systemic AL amyloidosis^30^. Native T1 mapping offers a promising alternative for LGE, especially in patients with severe renal dysfunction in whom gadolinium contrast is contraindicated. However, because of the lack of reproducibility for different scanners, a normal T1 value should be established in each institution^28,30,31^.

### Laboratory testing of circulating proteins

If amyloidosis is suspected, then testing for paraproteins should be performed as part of the workup for diagnosis/exclusion of AL amyloidosis. The traditional approach is to perform serum and urine protein electrophoresis (SPEP and UPEP), with the finding of an M-spike suggestive of a possible monoclonal protein. However, these tests have low sensitivities for AL amyloidosis and should be complemented by immunofixation of the serum and urine and the calculation of the serum FLC (sFLC) ratio (ratio of free kappa over lambda light-chain levels). Mass spectrometry of blood and urine may replace serum and urine immunofixation to further improve sensitivity^32^. In AL amyloidosis, the sFLC assay will show an abnormal kappa–lambda ratio that can additionally be used to track responses to therapy. Notably, an abnormal sFLC ratio must be examined in the context of each patient because a significant subset of individuals may have low-grade, non-specific circulating light-chains, and it is not uncommon (20 to 40% of cases) for patients with ATTR CA to also have an unrelated monoclonal gammopathy of undetermined significance (defined as an overgrowth of clonal plasma cells <10% along with a serum monoclonal protein <3 g/dl but without end-organ damage) without overproduction of light-chains^33^. Therefore, hematology input can be very helpful in understanding the exact cause of CA in these cases. In general, an abnormally low kappa–lambda ratio of <0.26 is suggestive of a monoclonal lambda light-chain process, while an abnormally high kappa–lambda ratio of >1.65 is suggestive of a monoclonal kappa light-chain process. Because light-chains are excreted by the kidney, the absolute serum levels of both kappa and lambda will be elevated in renal dysfunction but the ratio should remain normal^21^. It is important to recognize that while circulating TTR is detectable, tetramer stability and overall “burden” of abnormal TTR deposition cannot be quantified by routine measurements of circulating pre-albumin.

Recently, a proof-of-concept study showed that an expert-independent machine learning prediction model for CA relying on routinely determined laboratory parameters was able to generate a CA-related HF profile that may help increase disease awareness among physicians and improve timely diagnosis^34^. With the continuous improvement in machine learning techniques and the integration of imaging and laboratory parameters, this approach holds promise for the future.

### Nuclear imaging

Myocardial bone-avid radiotracer (such as ^99m^technetium pyrophosphate [^99m^Tc-PYP], 3.3-diphosphone-1,2-propanodicarboxylic acid [^99m^Tc-DPD], or hydroxymethylene diphosphonate [^99m^Tc-HMDP]) uptake is highly specific for ATTR CA. The exact mechanism underlying the myocardial retention of these tracers is unknown but may be due to the presence of cardiac tissue microcalcifications that are more common in ATTR amyloidosis than in AL amyloidosis^4^. Multiple guidelines support a non-biopsy diagnosis of ATTR CA using ^99m^Tc-PYP/DPH/HMDP scintigraphy but emphasize both its use in the appropriate clinical context (e.g. patients with high pre-test likelihood) and the crucial need to first rule out AL amyloid cardiomyopathy with the combination of SPEP and UPEP with immunofixation and sFLC ratio^33^. In our center, laboratory testing and a ^99m^Tc-bone scintigraphy are often planned together in the work up for CA because of logistic reasons and physician preference (Figure 2, Step 2). Technetium-labeled cardiac scintigraphy scans with bone-seeking tracers are evaluated using either a qualitative visual grading score (grade 0 means no myocardial uptake and normal rib uptake while grade 3 means myocardial uptake greater than rib uptake) or a semi-quantitative method using the heart to contralateral lung (HCL) uptake ratio or heart to whole body ratio. Several studies have shown that there is a significant myocardial uptake in ATTR CA (grade 2 or 3) but no to mild uptake in AL CA (grade <2)^21,35,36^. A large multicenter study assessed the diagnostic accuracy of all three previously mentioned tracers in 1,217 patients with suspected CA and found that grade 2 or 3 myocardial radiotracer uptake and the absence of a monoclonal protein in serum or urine (based on serum and urine immunofixation and sFLC assay) had a specificity and positive predictive value for ATTR CA of 100%, albeit with a sensitivity of 70%^37^. If there is any inconsistency between different tests, further investigation (e.g. with endomyocardial biopsy) is necessary.

Lately, there is increasing interest in using thioflavin-analogue tracers, such as ^18^F-florbetapir, ^18^F-florbetapen, ^18^F-flutemetamol, and ^11^C-labeled Pittsburg compound-B. These radiotracers are promising for the quantification and assessment of response to therapy in the future, but further data are needed to define their accuracy and additive value to the care of patients with suspected CA.

### Genetic testing

Differentiating the ATTRwt variant and the ATTRm variant is done by testing the *TTR* gene for a mutation^38^. The ATTRm variant can manifest as a polyneuropathy, cardiomyopathy, or a mixed phenotype. The most common mutation in the United States, present in 3.4% of African Americans, is V122I, in which there is an isoleucine substitution for valine at the 122^nd^ amino acid position^4^. The second most common mutation in the United States, T60A, is seen in patients of Irish descent^39^. Outside the US, the V30M is the most frequent pathogenic mutation and is especially present in endemic areas in Portugal, Sweden, Japan, and South America^40^.

Beyond diagnostics, genetics may also help to improve therapy-related outcomes in AL amyloidosis. Fluorescence in situ hybridization genetics show a t(11;14) variation in approximately 50% of patients. Patients with this translocation appear to have lower response rates to bortezomib-containing regimens but may see a greater benefit from an alkylating agent than those without the translocation^41,42^.

### Endomyocardial or fat pad biopsy

When amyloid fibrils infiltrate the heart, deposition is most commonly diffuse with amyloid depositions surrounding each myocyte. Thus, endomyocardial biopsy is 100% sensitive for the diagnosis of CA^43^. However, it should be noted that the myocardial deposits may be heterogeneous, so multiple biopsies are recommended, and each biopsy carries a small risk of right ventricular perforation (1%)^44^. Although birefringence under polarized light microscopy is considered histopathologically diagnostic for amyloidosis, Congo Red staining of the biopsy tissue alone is sometimes not sufficient and other methods need to be used^45^. In particular, Congo Red staining requires a skilled and experienced pathologist who may not be available in all institutions to accurately visualize the apple-green birefringence under polarized light microscopy. Some pathology laboratories rely on fluorochrome dyes such as thioflavin S staining, which can be a more sensitive technique even though it is not a specific stain for amyloid fluorescence. Moreover, subtyping the type of amyloidosis by the pathologist is absolutely crucial. Subtyping can be performed by immunohistochemistry and/or mass spectrometry for accurate identification of the precursor protein type^18,46^.

While commonly performed, fat pad biopsy has diagnostic limitations, and a negative fat pad biopsy does not rule out amyloidosis. The sensitivity is 60–80% sensitive in AL amyloidosis but only 14% in ATTRwt. Nevertheless, if amyloid is present in a fat pad biopsy and clinical features of CA are evident in imaging studies, endomyocardial biopsies are no longer necessary for diagnosis.

### Right heart catherization

Hemodynamic analysis is important for managing HF in CA but is less useful in the diagnosis of amyloidosis. Right heart catheterization is nonspecific but often shows restrictive filling patterns that can inform disease severity and prognosis. Cardiac output can be preserved but is more commonly low in patients with progressive HF symptoms.

## Advances in the treatment of cardiac amyloidosis

Until recently, treatment options for AL CA and ATTR CA were limited. However, several therapeutics have been recently approved to target the amyloid deposits directly, and multiple novel therapeutics are in development, holding the promise to revolutionize the clinical management of these conditions. Figure 1 and Table 3 present different targets of therapy as well as practical aspects of each treatment. The aims of therapy for ATTR and AL CA are different. Systemic AL amyloidosis requires treatment targeted against the abnormal plasma cell clone, whereas therapies for ATTR CA target the production and destabilization of the TTR molecule. The majority of these treatments interrupt particular steps during amyloidogenesis^3^, while some use monoclonal antibodies to target the removal of existing deposits. A multistep approach will likely be the most optimal way to manage both AL CA and ATTR CA, and meticulously conducted clinical trials are necessary to decide which approach is best^3^. Additionally, treatment of CA must include the management of restrictive cardiomyopathy with preserved or reduced ejection fraction in addition to treating the amyloid disease.

**Table 3.**
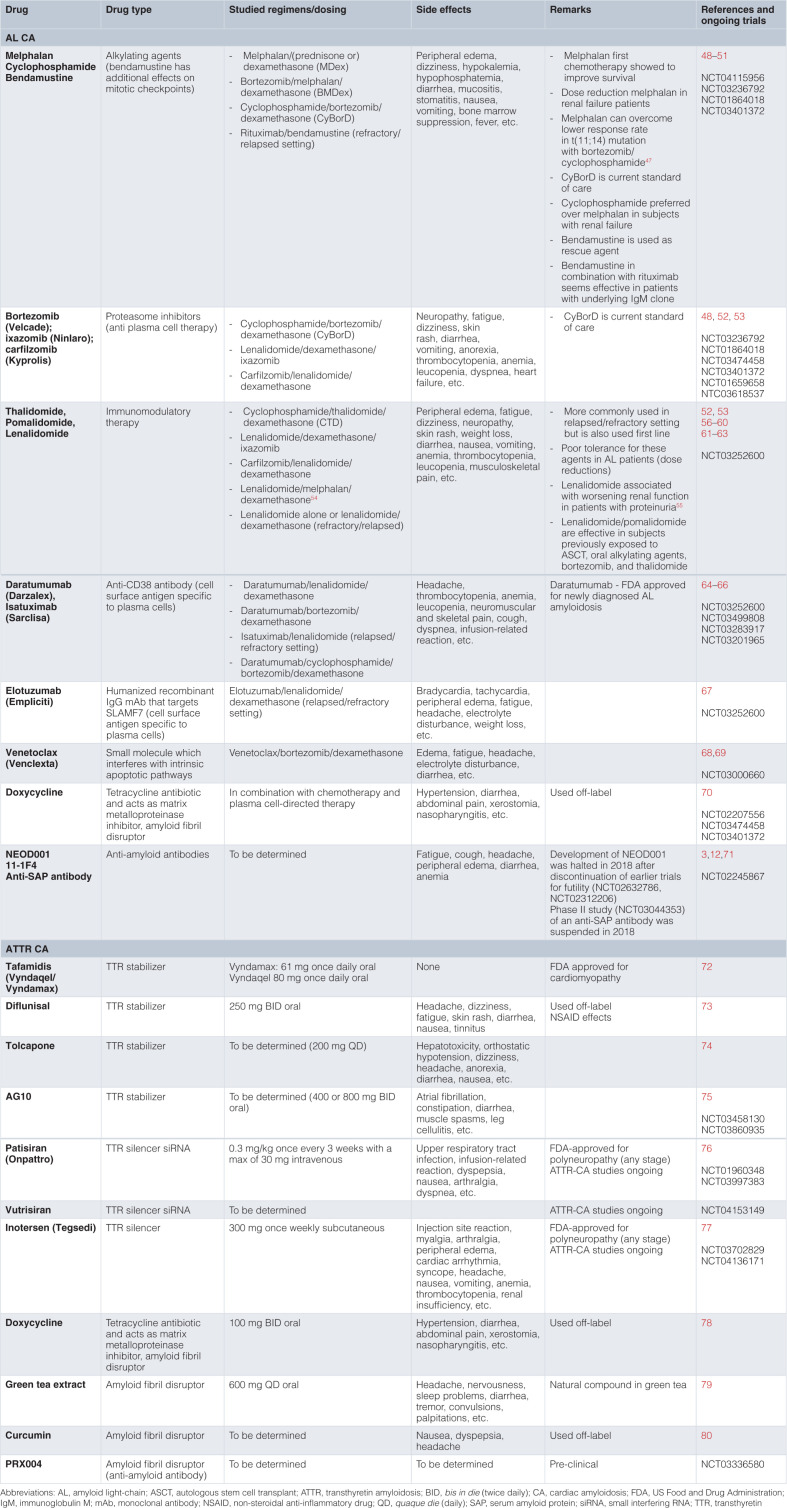
Therapies for current therapies for AL and ATTR CA.

### Light-chain amyloidosis

Unusual serum light-chains compel disease progression in AL CA, and thus the main focus during treatment is maximum reduction of pathogenic light-chain levels. The majority of studies show that overall survival and the degree of hematologic response are firmly connected, highlighting the necessity to change the treatment approach if light-chain levels are not suitably reduced by the existing regimen^12^.

Common light-chain suppressive therapies, used alone or in combination, are alkylators, proteasome inhibitors, steroids, immunomodulatory drugs (thalidomide-related drugs), antibodies directed to cell surface antigens on plasma cells, and autologous stem cell transplantation (ASCT). However, there are very few controlled studies investigating the most appropriate treatment regimen for different settings, and the design of individual treatment strategies relies on expert opinions mostly based on retrospective trials^47^. The first regimen to improve outcomes in AL amyloidosis was a combination of melphalan and a steroid. Owing to the poor outcomes with standard chemotherapy (median survival of 6 to 18 months), high-dose melphalan followed by ASCT was investigated and showed a significant improvement in both short- and long-term survival^81–84^. However, there is conflicting evidence regarding the role of ASCT for the treatment of amyloidosis. Trials that suggest superiority of ASCT over standard chemotherapy are mostly retrospective case(-control) studies, and these studies are criticized for the fact that patients who underwent ASCT were overall healthier than those who did not. The only randomized trial to date showed no superiority of ASCT over standard chemotherapy, but this trial was published more than 10 years ago and the chemotherapy arm is not representative of current “standard” chemotherapy/immunotherapy regimens^85^. Currently, most patients with a new diagnosis of AL amyloidosis are started on a regimen combining the alkylator cyclophosphamide, the proteasome inhibitor bortezomib, and the steroid dexamethasone (CyBorD). This regimen yielded an overall hematologic response rate of 60% in a European study of 230 patients and even higher hematologic response rates up to 94% in smaller studies^86–88^. Other proteasome inhibitors, which cause cell cycle arrest and cellular apoptosis, especially in proliferating malignant cells, such as carfilzomib and the oral agent ixazomib have shown promise but because of their treatment toxicities are not typically chosen as first-line therapy^12,89,90^. Immunomodulatory agents (e.g. thalidomide, pomalidomide, and lenalidomide), which inhibit pro-inflammatory cytokine production and angiogenesis and induce apoptosis, are also commonly used in the treatment of AL amyloidosis, though rarely as first-line therapies^3^. For example, pomalidomide was shown to be effective in relapsed and refractory AL amyloidosis patients^91^. Of note, these therapies are associated with an increased thrombosis risk.

One of the most revolutionary advancements in AL amyloidosis management in the last several years comes from the ANDROMEDA phase III trial (NCT03201965), which demonstrated promising results of up-front subcutaneous daratumumab added to CyBorD. Daratumumab is a monoclonal antibody directed against CD38, a cell surface-specific antigen on myeloma cells, that can lead to plasma cell death through antibody-dependent and complement-dependent cytotoxicity. In the ANDROMEDA trial, daratumumab in combination with CyBorD significantly improved the rate of hematologic complete response compared to CyBorD alone (53.3% versus 18.1%, odds ratio [OR] 5.1, 95% confidence interval [CI] 3.2 to 8.2, *P* <0.0001)^92^. Approximately 20% of AL amyloidosis patients have advanced cardiac involvement at diagnosis (stage IIIb), and treatment of these patients remains an unmet need. Ideally, these patients need a very rapidly acting, safe regimen^93^. The safety profile and rapidity of action of subcutaneous daratumumab make this agent very appealing in this setting, and a phase II trial is currently ongoing (NCT04131309). Other monoclonal antibodies of interest are isatuximab and elotuzumab (directed against SLAM7)^3,94^. Notable toxicities of monoclonal antibodies are infusion reaction and hypogammaglobulinemia^12^. Venetoclax** is a small molecule that binds with high affinity to intrinsic pro-apoptotic proteins and induces apoptosis^95,96^ and is used as a second- or third-line therapy or used in conjunction with other standard therapies.

Recently, it was shown in a phase II trial that bendamustine, an alkylating agent with additional effects on mitotic checkpoints, in combination with dexamethasone can prolong survival in patients with relapsed or refractory AL amyloidosis and is relatively well tolerated, so this regimen can compete with other drug regimens^97^.

Besides light-chain suppressive therapies, pharmacologic strategies focused at the removal of amyloid deposits are also of interest in the treatment of AL amyloidosis. Doxycycline, a tetracycline antibiotic,** may mitigate the cardiotoxic effects of light-chain amyloid fibril deposition in the extracellular cardiac environment. In a retrospective cohort study of 103 patients with AL CA, 24-month survival improved from 40% to 82% by administering doxycycline along with chemotherapy, while cardiac response to therapy improved threefold^70^. Although this early study is promising, rigorous clinical trial data are currently lacking^98^.**

Animal studies have suggested that exogenous administration of anti-amyloid antibodies (11-1F4, NEOD001) may expedite the clearance of amyloid deposits by phagocytes^70,99,100^. In a human study of patients with cardiac involvement, 11-1F4 led to a statistically significant improvement in global longitudinal strain after 12 weeks of follow up^3^. NEOD001 is an antibody with the potential to both bind to soluble aggregates of amyloid protein and help in the clearance of amyloid deposits in tissues. Despite promising results in a phase I/II study, a phase III study of NEOD001 treatment failed to meet its endpoint and a second phase III trial was stopped for futility^71,100,101^. Another treatment approach targets SAP, a glycoprotein that stabilizes and protects amyloid fibrils from degradation^40^. In a phase I trial that administered both an agent to deplete circulating SAP and a monoclonal antibody targeting SAP, there was modest evidence of decreased amyloid deposits^102^. However, a phase II study (NCT03044353) of an anti-SAP antibody specifically focusing on CA (both ATTR and AL CA) was suspended in 2018 owing to a “change in benefit/risk profile”^12^. Therefore, additional studies are needed to determine the efficacy and practicality of an anti-SAP treatment approach.

### Transthyretin amyloidosis

Previously, ATTR CA could be treated only with organ transplantation, but now pharmaceutical therapies can slow or halt ATTR CA progression and favorably affect clinical outcome. Early recognition remains essential to afford the best treatment efficacy.

Orthotopic liver transplantation was first proposed in 1990 as a potentially curative treatment for ATTRm-related polyneuropathy. Liver transplantation removes the source of mutated TTR molecules, so is not a treatment option for patients with ATTRwt, and was shown to prolong survival in ATTRm patients. However, tissue accumulation of TTR fibrils can continue after liver transplantation, likely because existing TTR amyloid fibrils promote subsequent deposition of TTRwt^40,103^. Combined heart and liver transplant is feasible in selected patients with ATTRm cardiomyopathy, and small studies suggest that dual organ transplant is associated with a better prognosis than cardiac transplant alone, although this remains a point of controversy^40,104,105^. Based on current insights and newly available therapies to slow disease progression, heart transplant alone is likely sufficient to treat advanced forms of ATTR CA^40^.

TTR tetramer stabilizers (diflunisal, tafamidis, AG10, tolcapone) have emerged as a novel class of therapeutics for ATTR and stabilize the tetramers so that the formation of misfolded monomers is inhibited. The non-steroidal anti-inflammatory drug (NSAID) diflunisal was one of the first TTR tetramer stabilizers identified^106^. A phase I study showed that diflunisal can stabilize TTR tetramers and prevent amyloid fibril formation *in vitro*^40^. However, a small single-arm open-label study with an average follow up of <1 year could not show an effect on cardiac dysfunction^107^. Diflunisal appears to be safe, but the adverse effects from chronic NSAID treatment are of concern, especially in a HF population. The small molecular ligand tafamidis effectively and selectively binds TTR without NSAID influence. It dose-dependently inhibits wild-type TTR amyloidogenesis as well as provides stability to V30M and V122I with similar potency and efficacy^108^. In the ATTR-ACT trial, a phase III trial of 441 patients with wild-type and hereditary ATTR CA, tafamidis led to a reduction in all-cause mortality (hazard ratio [HR] 0.70, 95% CI 0.51 to 0.96), with a number needed to treat for the combined endpoint of all-cause mortality and cardiovascular-related hospitalization of 7.5^72^. The Kaplan-Meier survival curves started diverging after 18 months of treatment, which is in agreement with the concept of tafamidis being a disease-modifying drug^40^. It is currently unknown if tafamidis is also effective in patients with advanced amyloid disease (NYHA class IV HF), since these patients were excluded from the study.

AG10 is a selective, oral TTR stabilizer that mimics a protective *TTR* mutation and binds to wild-type TTR with higher affinity than tafamidis or diflunisal do. Recently, the positive results of a phase II trial with AG10, demonstrating that the drug is well tolerated and can achieve near-complete stabilization of TTR, has led to the initiation of a phase III trial (NCT03860935)^75^. Tolcapone, an FDA-approved adjuvant treatment for Parkinson’s disease, binds to the thyroxine-binding pocket at the TTR dimer–dimer interface with higher affinity than tafamidis and is a stronger aggregation inhibitor than tafamidis^109^. However, despite its high potential for TTR stabilization, tolcapone should be used with caution because of a serious risk of acute fulminant liver failure^21^.

TTR synthesis inhibitors** (patisiran and inotersen) work by targeting the TTR mRNA^3^. Patisiran, a small interfering RNA, targets the *TTR* gene^76^. It is delivered to the cytoplasm via endosomal endocytosis of the lipid nanoparticle, which is given as an intravenous infusion. Patisiran binds to the RNA-induced silencing complex, inducing the separation of the two RNA strands and enabling the antisense strand to bind to TTR mRNA. The substrate is cleaved and is subsequently degraded, resulting in TTR protein level reduction^110^. Patisiran was approved in the US and Europe for the treatment of ATTRm-related polyneuropathy after the positive findings regarding neurological status in the phase III APOLLO trial^76^. Based on a substudy of patients with evidence of CA in the APOLLO trial, it seems that patisiran may halt or reverse the progression of cardiac manifestations of ATTRm^111^. Indeed, a separate small study demonstrated that patisiran could reduce extracellular volume measured by cardiac magnetic resonance and thus provides evidence for ATTR cardiac amyloid regression^112^. The purpose of the ongoing APOLLO-B study (NCT03997383) is to specifically evaluate the efficacy of patisiran on functional capacity, hospitalizations, and mortality in patients with ATTR CA. Inotersen, a second-generation antisense oligonucleotide that binds to a region of TTR mRNA, elicits enzymatic degradation and reduced protein expression in both ATTRwt and ATTRm^21,113^. In the NEURO-TTR trial, which was a multicenter, randomized placebo-controlled phase III trial, inotersen demonstrated a significant improvement in neurologic functioning and quality of life compared to the placebo arm to the extent that inotersen has now received FDA approval for patients with ATTRm-related polyneuropathy^77^. However, this study was not powered to measure the effects on cardiac disease, leaving the effects of inotersen on CA unclear. An open-label study is currently investigating the effect of inotersen on cardiac structure changes in patients with both wild-type and hereditary ATTR CA (NCT03702829).

As discussed for AL amyloidosis, antibodies directed at SAP are of interest for treating ATTR CA, but the efficacy of this treatment approach in CA has yet to be demonstrated. After discontinuation of a phase II and a phase I trial (https://adisinsight.springer.com/drugs/800038353), there are no evaluations of anti-SAP antibody ongoing^40^. A monoclonal antibody designed to target TTR amyloid deposits (PRX004) did enter clinical evaluation with an ongoing phase I study (NCT03336580) in ATTRm patients.

### Cardiac-specific treatment

Although management of the amyloid disease is the backbone of treatment for CA, treating cardiac dysfunction in parallel is of great importance. The natural history of CA includes progressive HF, complicated by arrhythmias and conduction system disease^4^.

***Medical therapy.*** The mainstay of medical therapy is often volume management. Keeping patients in a euvolemic state can be very challenging, since both excessively low and high filling pressures are not well tolerated^21^. Patients with CA can be prone to the challenging combination of hypotension with concomitant volume overload due to diastolic dysfunction, coexisting renal or hepatic dysfunction leading to reduced serum albumin concentration, and concomitant autonomic dysfunction from amyloid deposition leading to postural hypotension. Salt restriction and appropriate diuretic doses are essential for most patients. If symptomatic hypotension limits the ability to achieve euvolemia, midodrine can be useful. Classic HF drugs are not as useful in the setting of CA. For example, beta-blockers and angiotensin-converting enzyme inhibitors are not well tolerated or not effective in CA, most likely because of dependence of the cardiac output on heart rate and the tendency for orthostatic hypotension^114^. Moreover, non-dihydropyridine calcium channel blockers bind to amyloid fibrils and are relatively contraindicated owing to the risk of profound hypotension and syncope^21,115–117^. Digoxin is usually avoided in CA because of concerns of increased risk of toxicity; however, it may be used with caution for rate control in atrial fibrillation given its lack of negative inotropy^21^.

***Rhythm management.*** Atrial arrhythmias are common in patients with amyloidosis^118^. Maintenance of normal sinus rhythm is preferable owing to the importance of atrial contribution to cardiac output. Importantly, anticoagulation in patients with atrial fibrillation, and even in patients with normal sinus rhythm but poor atrial function, is important because of the high risk of thromboembolic complications in amyloid patients. It is therefore recommended to perform transesophageal echocardiography before elective cardioversion in all patients with amyloidosis, even if they have been on consistent therapeutic anticoagulation^119^. Pacemakers are indicated for heart block and symptomatic bradycardia, which is more prevalent in ATTR CA than in AL CA^120,121^. The role of intracardiac defibrillators, however, is controversial. Traditionally, implantable cardioverter defibrillator (ICD) placement was considered contraindicated in patients with CA owing to poor overall prognosis, a purported lack of successful resuscitation with ICD shocks in CA patients with ventricular arrhythmias, and many episodes of sudden cardiac death caused by bradyarrhythmias^121^. In general, though, cardiologists currently follow traditional ICD criteria for primary and secondary prevention, since the life expectancy of most CA patients now exceeds 1 year thanks to advancements in therapy^122,123^.

***Advanced heart failure therapies.*** Because of the advancements in treatment of ATTR and AL amyloidosis, advanced HF therapies are increasingly considered in managing CA. Generally, CA patients are not considered suitable candidates for durable LV assist devices (LVAD) owing to a small LV cavity impeding outflow cannula placement or because of biventricular involvement. Recent case series, however, have shown that in carefully selected patients with CA, LVAD (and especially a total artificial heart) is feasible and may be a lifesaving therapy or bridge to transplantation^124–126^.

Early reports of cardiac transplantation for amyloidosis were overall discouraging, but there has been a significant improvement in outcomes in the last decade, with outcomes now matching those of non-CA restrictive cardiomyopathies. This likely reflects better light-chain suppressive therapies and a higher proportion of transplants for ATTR amyloidosis^127^. Patients with AL amyloidosis, more than ATTR amyloidosis, may have extra-cardiac involvement that would exclude heart transplantation or lead to worse post-transplant outcomes. Nevertheless, the relatively high mortality on the transplantation list has prompted their higher priority in the 2018 revision of the U.S. heart transplant policy (Status 4)^128,129^. SAP labeled with radioactive ^123^iodine has been used as a tracer to determine the extent and distribution of amyloid deposits by scintigraph and turnover studies^130,131^. Similarly, it has been shown that radioiodinated p5+14 can bind specifically to amyloid deposits in tissues. Currently, a single-center phase I study is evaluating if ^124^I-p5+14 positron emission tomography/computed tomography can be used as an imaging technique to identify and quantify whole body and/or organ amyloid deposition in systemic amyloidosis (NCT03678259). Recent data show that in AL CA patients who are young and healthy enough to receive chemotherapy, heart transplant followed or preceded by chemotherapy and eventual hematopoietic stem cell transplant is a reasonable treatment plan. Most heart transplant programs require that AL amyloidosis patients demonstrate a reasonable response to chemotherapy and are a candidate for bone marrow transplantation in order to be considered for heart transplantation. The most common cause of death after heart transplant/ASCT in AL amyloidosis is the recurrence of light-chain production with amyloid deposition in the heart and other organs^129^. In contrast, patients with ATTR CA are more likely to have isolated cardiac involvement, so require fewer systemic therapies, and have better survival following heart transplantation^129^. ATTR is predominantly a disease of the elderly, and progression is usually slow, so significant damage to the transplanted heart is unlikely. Dual heart-liver transplants were originally proposed for ATTR CA because of the hepatic production of TTR but are now seldomly performed.

## Conclusion

CA is caused, in the vast majority of cases, by either TTR or AL amyloidosis. Its prevalence is more common than previously thought, especially among patients with HFpEF and aortic stenosis. If there is a clinical suspicion of CA, focused echocardiography, SPEP and UPEP with immunofixation and sFLC ratio, and/or ^99m^Tc cardiac scintigraphy are the preferred initial diagnostic tests. In some cases, more advanced diagnostic techniques are necessary. Treatment depends on the underlying form of amyloidosis and targets the formation and deposition of amyloid fibrils. In case of AL CA, close collaboration with a hematologist is necessary. Besides treating the amyloid disease, these patients often need treatment of HF and other cardiac symptoms. In advanced stages, cardiac assist devices and transplantation can be pursued, especially in the case of ATTR CA. The fields of diagnosis and management of both AL CA and ATTR CA are rapidly evolving. Future research investigating optimal management strategies for AL CA and ATTR CA and techniques to follow cardiac response to therapy will further improve the management of CA and increase the quality of life of CA patients.

## Abbreviations

^99m^Tc-PYP, ^99m^Technetrium pyrophosphate bone scintigraphy;^ 99m^Tc-DPD, ^99m^Technetrium 3.3-diphosphone-1,2-propanodicarboxylic acid bone scintigraphy; ^99m^Tc-HMDP, ^99m^Technetrium hydroxymethylene diphosphonate bone scintigraphy; AL, amyloid light-chain; ASCT, autologous stem cell transplantation; ATTRwt, wild-type transthyretin amyloidosis; ATTRm, hereditary (or mutant) transthyretin amyloidosis; CA, cardiac amyloidosis; HF, heart failure; HFpEF, heart failure with preserved ejection fraction; LGE, late gadolinium enhancement; LV, left ventricle; MRI, magnetic resonance imaging; NSAID, non-steroidal anti-inflammatory drug; NYHA, New York Heart Association Class; TTR, transthyretin; sFLC ratio, serum free light-chain ratio; SPEP, serum protein electrophoresis; UPEP, urinary protein electrophoresis
